# Ceruloplasmin, NT-proBNP, and Clinical Data as Risk Factors of Death or Heart Transplantation in a 1-Year Follow-Up of Heart Failure Patients

**DOI:** 10.3390/jcm9010137

**Published:** 2020-01-03

**Authors:** Ewa Romuk, Wojciech Jacheć, Ewa Zbrojkiewicz, Alina Mroczek, Jacek Niedziela, Mariusz Gąsior, Piotr Rozentryt, Celina Wojciechowska

**Affiliations:** 1 Department of Biochemistry, Faculty of Medical Sciences in Zabrze, Medical University of Silesia, 40-055 Katowice, Poland; 2 Second Department of Cardiology, Faculty of Medical Sciences in Zabrze, Medical University of Silesia, 40-055 Katowice, Poland; wjachec@interia.pl (W.J.); wojciechowskac@wp.pl (C.W.); 3 Department of Toxicology and Health Protection, Faculty of Health Sciences in Bytom, Medical University of Silesia, 40-055 Katowice, Poland; ezbrojkiewicz@op.pl (E.Z.); alina.mroczek@wp.pl (A.M.); prozentryt@sum.edu.pl (P.R.); 4 3rd Department of Cardiology, Faculty of Medical Sciences in Zabrze, Medical University of Silesia, Silesian Centre for Heart Disease, 41-800 Zabrze, Poland; jacek.niedziela@gmail.com (J.N.); m.gasior@op.pl (M.G.)

**Keywords:** ceruloplasmin, NT-proBNP, heart failure

## Abstract

We investigated whether the additional determination of ceruloplasmin (Cp) levels could improve the prognostic value of N-terminal pro-B-type natriuretic peptide (NT-proBNP) in heart failure (HF) patients in a 1-year follow-up. Cp and NT-proBNP levels and clinical and laboratory parameters were assessed simultaneously at baseline in 741 HF patients considered as possible heart transplant recipients. The primary endpoint (EP) was a composite of all-cause death (non-transplant patients) or heart transplantation during one year of follow-up. Using a cut-off value of 35.9 mg/dL for Cp and 3155 pg/mL for NT-proBNP (top interquartile range), a univariate Cox regression analysis showed that Cp (hazard ratio (HR) = 2.086; 95% confidence interval (95% CI, 1.462–2.975)), NT-proBNP (HR = 3.221; 95% CI (2.277–4.556)), and the top quartile of both Cp and NT-proBNP (HR = 4.253; 95% CI (2.795–6.471)) were all risk factors of the primary EP. The prognostic value of these biomarkers was demonstrated in a multivariate Cox regression model using the top Cp and NT-proBNP concentration quartiles combined (HR = 2.120; 95% CI (1.233–3.646)). Lower left ventricular ejection fraction, VO_2_max, lack of angiotensin-converting enzyme inhibitor or angiotensin receptor blocker therapy, and nonimplantation of an implantable cardioverter-defibrillator were also independent risk factors of a poor outcome. The combined evaluation of Cp and NT-proBNP had advantages over separate NT-proBNP and Cp assessment in selecting a group with a high 1-year risk. Thus multi-biomarker assessment can improve risk stratification in HF patients.

## 1. Introduction

Systolic heart failure (HF) is a complex disease caused by reduced ejection fraction of the left ventricle, often leading to the worsening of symptoms and poor quality of life, despite proper diagnosis and treatment according to current guidelines. All-cause mortality in these patients remains high and heart transplantation is a therapeutic option in end-stage HF. Adverse outcomes for HF patients are associated with many contributing factors. Stratification of risk factors is a great challenge in out-patient clinic cohorts, in which patients still undergo significant mortality and morbidity, despite stable HF. Different clinical and laboratory parameters can be helpful to identify patients at higher risk of adverse outcomes. Biological markers reflecting several pathophysiological abnormalities of HF have become powerful and convenient noninvasive tools for the stratification of HF patients [1,2,3]. Brain natriuretic peptide (BNP) and N-terminal pro-BNP (NT-proBNP) are secreted by cardiomyocytes in response to hemodynamic overload or neurohormonal disturbances. In clinical practice, NT-proBNP is recommended as a marker over BNP, because of its longer plasma half-life and lower levels of biological variation. NT-proBNP is the best-known diagnostic biomarker [4]. The usefulness of NT-proBNP for risk stratification varies depending on the stage of HF, time of assessment (onset of hospitalization, pre-discharge, or out-patient clinic evaluation), and duration of follow-up. However, there is no conclusive evidence that plasma NT-proBNP concentration is a guide for more effective therapy [5,6,7,8]. Ceruloplasmin (Cp) is an acute-phase reactant that is synthesized and secreted by the liver and monocyte/macrophages. It is elevated in conditions of acute inflammation. Cp contains seven copper atoms per molecule, participates in copper transport and metabolism, and has ferroxidase activity [9,10]. Furthermore, Cp is involved in the modulation of coagulation and angiogenesis and the inactivation of biogenic amines [11,12]. It is possible that increased levels of Cp may decrease available plasma NO, thus increasing reactive oxygen species formation and oxidative cell injury [13]. Several recent reports have indicated that Cp levels are elevated in patients with heart failure, regardless of its etiology [14,15,16].

Different pathobiological processes are involved in heart failure; thus, it is not surprising that single biomarkers, even natriuretic peptides, fail to predict all risks associated with HF.

The aim of this study was to examine the prognostic value of clinical factors, with special consideration of Cp, in a large cohort of HF patients and to investigate whether the combination of Cp and NT-proBNP could provide additional prognostic information in HF patients in a 1-year follow-up.

## 2. Materials and Methods

### 2.1. Clinical Assessment

We analyzed data in a subgroup of patients included in the Prospective Registry of Heart Failure (PR-HF) and Studies Investigating Co-morbidities Aggravating Heart Failure (SICA-HF) studies described elsewhere [17]. A cohort of patients with chronic systolic HF were prospectively recruited from patients referred to our inpatients clinic as potential candidates for heart transplantation. The main inclusion criteria were reduced left ventricular ejection fraction (LVEF ≤ 40%) and symptomatic HF, despite pharmacological treatment according to the current published ESC guidelines, at least 3 months before inclusion. The exclusion criteria included acute myocardial infarction; pulmonary thromboembolism; constrictive pericarditis; infectious pericarditis; prior heart transplantation; noncardiac conditions resulting in an expected mortality of less than 12 months, as judged by the treating physician; and a history of alcohol abuse or known antioxidant supplementation. These criteria were fulfilled in the 1216 PR-HF and SICA-HF studies. We analyzed data from 741 participants (aged 48–59 years) who had completed clinical and laboratory assessments.

A detailed description of the clinical echocardiographic evaluation of patients included in the study has been presented elsewhere [18].

The primary outcome was a composite of death from all causes (nontransplant patients) or heart transplantation. In the case of heart transplantation, the endpoint was reached and the patient was not followed up further. Patients were followed for a year via direct or phone contact. In some cases, the exact data regarding patient death were obtained from family members or the national identification number database by dedicated research personnel. Prior to enrolment in the study, all participants provided written informed consent. The local ethics committee of Silesian Medical University approved the study protocol (NN-6501-12/I/04). All procedures were performed in accordance with the 1975 Declaration of Helsinki and its revision in 2008.

### 2.2. Biochemical Methods

Venous blood samples obtained at enrollment were processed, separated by centrifugation at 1500× *g* for 10 min, frozen at −70 °C, and partially stored at −70 °C until assayed. Serum protein, albumin, fibrinogen, CRP, alanine aminotransferase, aspartate aminotransferase, gamma-glutamyl-transferase (GGTP), alkaline phosphatase, bilirubin, and lipid parameters and serum iron, sodium, creatinine, glucose, and uric acid concentrations were measured by colorimetric methods (Cobas 6000 e501; Roche, Basel, Switzerland). Hemoglobin, leukocytes, and platelets were measured using a MEDONIC M32C analyzer (Alpha Diagnostics, Warsaw, Poland). NT-proBNP was measured using a chemiluminescence method (Cobas 6000 e501).

Serum Cp concentration was determined spectrophotometrically, according to the Richterich reaction with p-phenyl-diamine [19]. Cp catalyzes the oxidation of colorless p-phenylenediamine, resulting in a blue-violet dye. Twenty microliters of serum was added to the test sample, while 20 µL of serum and 200 µL of sodium azide solution were added to the control sample to stop the reaction. Then, 1 mL of p-phenylenediamine dihydrochloride in acetate buffer was added to both test and control samples. After a 15-min incubation, 200 µL of sodium azide was added to the test sample. Finally, after a 15-min incubation, the absorbance of test and control samples was measured at 560 nm using a PerkinElmer VICTOR-X3 plate reader. The samples were not previously thawed before Cp assays. The intra-assay coefficient of variation was 3.7% and the intra-assay precision was 4%.

### 2.3. Statistical Analysis

Study participants were divided into subgroups based on Cp concentration quartiles (Table 1). Moreover, two subgroups, firstly, both Cp and NT-proBNP in the top quartile and, secondly, remaining patients (Cp or NT-proBNP in I–III quartiles including patients with Cp in I–III quartiles and NT-proBNP in I–IV quartiles or NT-proBNP in I–III quartiles and Cp in I–IV quartiles), were also compared (Table 2). The Shapiro–Wilk test was used to evaluate the distribution of all continuous variables. Continuous data are presented as the median, with the first and fourth quartiles (because of non-normal distribution of the data). Categorical data are presented as absolute numbers and percentages. The Kruskal-Wallis ANOVA test was used to compare both continuous and categorical data.

Estimations of risk were performed using a Cox proportional hazards model. Only complete data were analyzed. All demographic; clinical; echocardiography; laboratory; medication; and Cp and NT-proBNP data, expressed as the top quartiles individually or as the combined top quartiles of Cp and NT-proBNP concentration, were included in a univariate Cox analysis. Variables with a value of *p* ≤ 0.05 in the univariate analysis were included in the multivariate analysis. Two multivariate analysis models were built. The first model was based on the top Cp and NT-proBNP concentration quartiles separately and the second model was based on the combined top quartiles of Cp and NT-proBNP concentrations.

The results of the Cox analysis are presented as relative risks, with 95% confidence intervals (CIs). Cumulative survival curves for all-cause death or heart transplantation were constructed as the time to endpoint occurrence, using the Kaplan–Meier method. Survival curves were compared among groups according to quartiles of Cp, quartiles of NT-proBNP and between groups presented in Table 2, using the log-rank test, as appropriate.

The odds ratio (OR) of achieving the endpoint for the top quartiles of Cp and NT-proBNP concentrations were calculated. The same calculations were performed for the combined top quartiles of Cp and NT-proBNP concentrations. The predictive value of these parameters was then compared.

Statistical significance was set at *p* < 0.05. Statistical analyses were performed using STATISTICA 13.1 PL software (StatSoft, Cracow, Poland).

## 3. Results

### 3.1. Baseline Characteristics of the Entire Study Population and Subgroups in Relation to Ceruloplasmin Concentration

The study group included 741 systolic HF patients, with a median Cp concentration of 28.7 mg% (range, 23.7–35.8). The cohort was divided into quartiles of serum Cp concentration as follows: group I, 184 (24.8%) patients with a Cp concentration range of 8.0–23.6 mg/dL; group II, 184 (24.8%) patients with 23.7–28.6 mg/dL Cp; group III, 187 (25.2%) patients with 28.7–35.8 mg/dL Cp; and group IV, 186 (25.1%) patients with the highest Cp concentration quartile of 35.9–81.0 mg/dL. One hundred and twenty-eight (17.42%) patients reached the combined endpoint (101 deaths, 27 heart transplantations). The overall mortality rate during the 1-year follow-up period was 13.76% and the heart transplantation rate was 3.64%. The demographic, clinical, and laboratory parameters of all patient groups and subgroups, divided according to quartiles of serum Cp concentration, are presented in Table 1.

Neither age, sex, BMI, nor duration of symptoms before enrollment differed between groups. LVEF was reduced to a greater extent in group IV. The percentage of patients with atrial fibrillation was higher in group IV, but the frequencies of coronary artery disease, hypertension, diabetes mellitus, and implantable cardioverter-defibrillators (ICDs) were similar between groups. Pharmacological treatments were comparable between groups in terms of the use of angiotensin-converting enzyme inhibitors (ACE-Is), angiotensin receptor blockers (ARBs), beta-blockers, mineralocorticoid receptor antagonists (MRAs), and statins, but loop and thiazide diuretics and digitalis were more frequently used by group III patients. If ACE-I or ARB treatment was analyzed, their use was the lowest in patients in the 4th Cp quartile.

The following laboratory parameters, assessed in serum samples, were different among groups: NT-proBNP, Cp, sodium, creatinine clearance, protein, fasting glucose, lipid parameters, uric acid, bilirubin, aspartate transaminase, alanine transaminase, alkaline phosphatase, and GGTP (Table 1). Characteristic of examined group with division according to ceruloplasmin and NT-proBNP concentration quartiles are presented in Table 2

### 3.2. Ceruloplasmin, NT-proBNP and Prognosis

#### 3.2.1. Univariate Cox Regression Analysis

All demographic, clinical, exercise capacity, echocardiography, laboratory parameter, comorbidity, and pharmacotherapy data presented in Table 1 were assessed as risk factors for all-cause death or heart transplantation in a 1-year follow-up.

In univariate Cox regression analyses, among others, the top quartiles of NT-proBNP concentration (hazard ratio (HR) = 3.221, 95% CI (2.277–4.556)), Cp concentration (HR = 2.086, 95% CI (1.462–2.975)), and combined Cp and NT-proBNP concentration (HR = 4.253, 95% CI (2.795–6.471) were associated with a higher risk of death or heart transplantation.

All variables that reached *p* < 0.05 in a univariate Cox regression analysis are presented in Table 3.

#### 3.2.2. Multivariate Cox Regression Analysis

In the first multivariate Cox regression model, after adjusting for other clinical and pharmacotherapeutic predictors, neither the top Cp concentration quartile nor the top NT-proBNP concentration quartile were significant predictors of unfavorable outcomes (Cp, HR = 1.511, 95% CI (0.980–2.330); NT-proBNP, HR = 1.287, 95% CI (0.815–2.033))

The results of the second multivariate Cox regression model, in which the top individual Cp and NT-proBNP concentration quartiles were replaced with the combined top quartiles of Cp and NT-proBNP concentrations, are presented in Table 3. In this model, an LVEF lower by 1 % (HR = 1.069, 95% CI (1.032–1.106)), a maximum measured VO_2_ lower by 1 mL/min/kg b.m. (HR = 1.113, 95% CI (1.048–1.181)), absence of an ICD (HR = 7.575, 95% CI (3.278–17.502)), and lack of ACE-I and/or ARB therapy (HR = 2.195, 95% CI (1.234–3.906)) remained significant predictors of unfavorable outcomes. Among the laboratory parameters measured, only the combined top quartiles of Cp and NT-proBNP concentrations was associated with a higher risk of all-cause death and HT in a 1-year follow-up (HR = 2.120, 95% CI (1.233–3.646)).

#### 3.2.3. Kaplan–Meier Survival Analysis and Endpoint Odds Ratios

**Figure 1:**
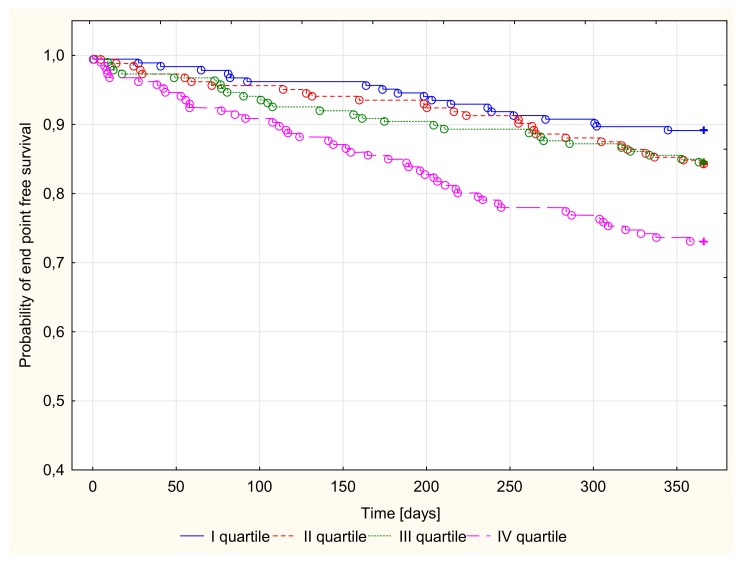
Probability of survival of time free of death or heart transplantation depending on quartiles of ceruloplasmin concentration in 1-year follow-up, *p* < 0.001.

**Figure 2:**
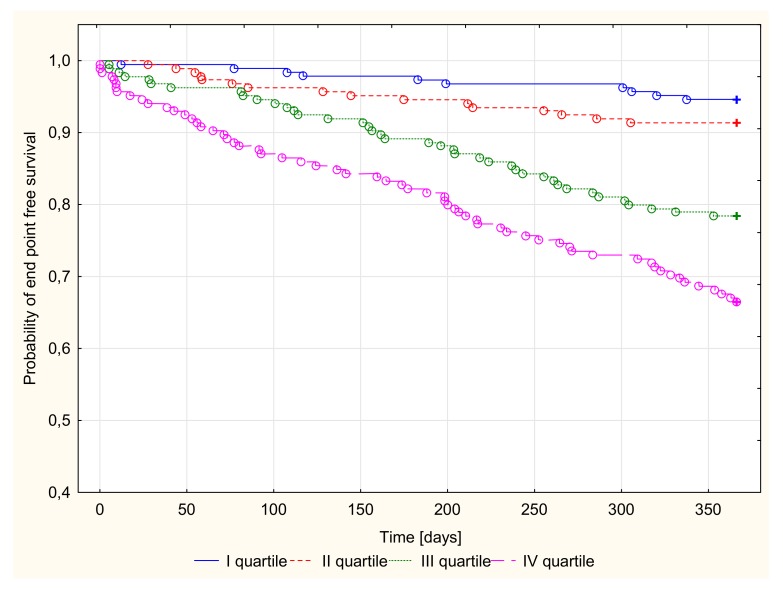
Probability of survival of time free of death or heart transplantation depending on quartiles of NT-proBNP concentrations in 1-year follow-up, *p* < 0.001.

**Figure 3:**
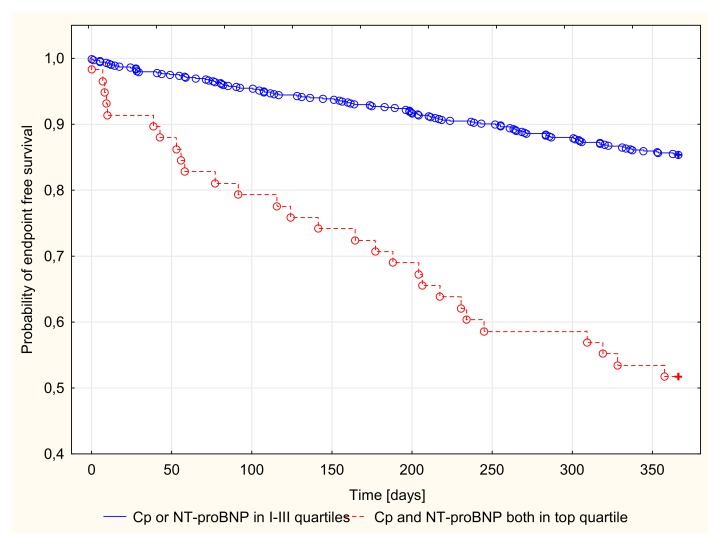
Probability of endpoint free survival in 1-year follow-up. Patients with Ceruloplasmin or NT-proBNP concentrations in I–III quartiles vs. both Cp and NT-proBNP in the top quartile, log rank *p* < 0.001.

There were 128 endpoints in groups I (20, 10.9%), II (29, 15.8%), III (29, 15.5%), and IV (50, 26.9%). Kaplan–Meier survival curves for the four groups according to Cp and NT-proBNP quartiles are presented in Figure 1 and Figure 2. Patients with both Cp and NT-proBNP concentrations in the top quartile were compared with the remaining patients (quartile I–III of Cp or NT-proBNP concentration), as shown in Figure 3.

A log-rank analysis revealed a significantly different probability of all-cause death or heart transplantation over time in patients stratified by quartiles of Cp or NT-proBNP concentration in the 1-year follow-up period (*p* < 0.001). After the stratification of patients based on the combination of CP and NT-proBNP concentration, patients with both Cp and NT-proBNP in the upper quartile had the highest probability of an endpoint occurrence (Table 4).

Detailed results for the top quartiles of Cp and NT-proBNP concentration, as well as the combination of the top quartiles of Cp and NT-proBNP concentrations, with the sensitivity and specificity of their predictive values, are presented in Table 4. For patients with a Cp concentration in the top quartile, the risk of death or heart transplantation was two-fold higher than in patients with Cp concentrations in quartiles I–III. Similarly, NT-proBNP concentration in the top quartile indicated approximately a 4-fold increase in the probability of an endpoint occurrence. The predictive values of Cp and NT-proBNP concentrations did not differ significantly (NT-proBNP vs. Cp, OR = 1.371, 95% CI (0.878–2.140)). The greatest prognostic value was seen for the combination of Cp and NT-proBNP concentrations in the top quartile, which was associated with more than a five-fold increased risk. Cp and NT-proBNP concentrations (both in the top quartile) showed a significantly higher predictive value than the top quartile of Cp (OR = 2.539; 95% CI (1.381–4.666)) or NT-proBNP (OR = 1.852; 95% CI (1.018–3.370)) concentrations individually (Table 4).

## 4. Discussion

There are many papers documenting the association between Cp and cardiovascular disease in clinical and experimental studies [20,21,22]. However, data confirming the effect of Cp concentration on prognosis in patients with HF are limited. This study intended to determine the clinical utility of a single baseline Cp measurement and other common risk factors as prognostic markers of all-cause mortality or heart transplantation in HF patients. We showed a significantly higher risk of all-cause death or heart transplantation in a 1-year follow-up of patients with Cp concentration in the top quartile. Similarly, patients with NT-proBNP concentration in the top quartile had a higher risk of endpoint occurrence. However, after adjustment for known clinical and laboratory parameters and treatments, neither NT-proBNP nor Cp remained significant predictors. Interestingly, the combination of elevated Cp and NT-proBNP concentrations (both in the top quartile) had greater specificity and sensitivity for endpoint prediction than CP or NT-proBNP concentrations alone. Other independent endpoint predictors were LVEF, peak VO_2_, ACE-I/ARB therapy, and prior ICD implantation. Although clinical assessment had a strong prognostic role, it is worth highlighting that peak oxygen consumption (peak VO_2_) rather than New York Heart Association class, should be used to estimate functional capacity. The utility of peak VO_2_ and other parameters of the Heart Failure Survival Score (ischemic heart disease, mean blood pressure, LVEF, heart rate, serum sodium, intraventricular conduction defect) for predicting prognosis and assessing candidacy for heart transplantation, have been documented across races and genders [23].

Recently, Paolillo et al. showed that the cut-off values of peak VO_2_ able to identify a 10% or 20% risk (in 10 years of follow-up) of unfavorable outcomes decreased over 20 years up to 2010, with similar cut-off values observed over this time period [24]. As a possible explanation, they suggested that the most effective treatment options were introduced to the guidelines by 2010, such that a similar risk level was observed in patients enrolled after 2010. In our study, patients were enrolled before 2010 and a decrease in peak VO_2_ by 1 mL/min/kg was associated with an 11% increase in the risk of endpoints in a 1-year follow-up. Lower values of the main echocardiographic parameter, LVEF, were associated with increased mortality or heart transplantation rate.

On the contrary, Lai et al. showed that, at initial presentation, LVEF did not have outcome-predictive power Additionally, they showed that the 12-month mortality risk in patients with LVEF ≥ 50% was similar to those with LVEF < 40% [7]. However, in this study, patients were hospitalized with acute HF, and therefore, LVEF data may reflect exacerbated heart function, rather than a chronic stable status.

Referring to guideline-based therapy, patient treatment in our study was considered to be optimized by the physicians [25]. Although we did not analyze the reasons for not using this treatment, in most cases there were contraindications to the use of this therapy. Moreover, we did not analyze the ACE-I/ARB dose, since even low-dose ACE-I/ARB therapy is superior to no one treatment as it decreases 1-year mortality rates [26]. Patient treatment may be a limitation of the study (see study limitation section). Beta-blockers were used in the majority of patients (97%). Notably, the percentage of patients with ICDs in our study was rather low (approximately 27%). The lack of ICD implantation was an independent risk factor of all-cause mortality (not only sudden cardiac death) and heart transplantation. Improved survival of patients with implanted ICDs has previously been observed in clinical trials [27]. ICD implantation was different between groups according to Cp concentration quartiles. In the highest Cp quartile the percentage of patients who did not receive ACE-I and /or ARB was the lowest. In our study, many of the analyzed laboratory parameters were risk factors of unfavorable outcomes only in univariable analyses. However, none of them were shown to affect mortality or heart transplantation after adjusting for other predictive factors. Only the combination of the top quartiles of NT-proBNP and Cp concentrations was useful for the prediction of unfavorable outcomes.

Previously, some investigations with various study designs have demonstrated the prognostic power of natriuretic peptide concentration [28,29,30]. Lai et al. reported that increased plasma NT-pro BNP level (≥11755 ng/L) was an independent predictor of 1- and 3-month mortality, but not of mortality in more extended follow-up [7]. Bettencourt and colleagues showed that an NT-proBNP concentration > 6779 pg/mL at admission was a weaker predictor of readmission or death than a post-treatment NT-proBNP concentration of 4137 pg/mL, with an 8% increase in the probability of death or readmission over 6 months per 1000 pg/mL of NT-proBNP [31]. Finally, the current ACC/AHA/HFSA Guideline for the Management of Heart Failure recommends the assessment of natriuretic peptide biomarkers on admission in acutely decompensated HF patients and before discharge, to establish a prognosis [32].

We evaluated NT-proBNP concentrations in stable, nonhospitalized patients and found that an NT-proBNP concentration > 3155pg/mL (upper quartile) did not have significant predictive value in a multivariate analysis in a 1-year follow-up. A comparison of our results with other studies is difficult because of different follow-up periods, endpoint definitions, and types of cohorts. Bayes-Genis et al. performed a serial assessment of NT-proBNP concentration in an outpatient group (patient decompensated, but not requiring emergency hospital admission) with reduced LVEF (27 +/− 9%). The percentage reduction in NT-proBNP concentration in the first four weeks (not baseline concentration) was a predictor of death and hospitalization during three months of follow-up [33].

Multiple biomarker strategies, involving a combination of NPs with other biomarkers, have been proposed to create more accurate predictive scores in HF [34]. Multimarker approaches combining NT-pro-BNP and Cp have been used to assess the risk of HF incidence and mortality in patients in the Atherosclerosis Risk in Communities (ARIC) study. In this population, the strongest associations of Cp were observed with HF and all-cause mortality. These associations persisted after adjusting for biomarkers known to have a role in HF prediction, such as NT-proBNP, troponin, and CRP [35]. Engstrom et al. also reported Cp as a risk factor for HF incidence in Caucasian men with a high risk of cardiovascular disease [36].

Elevated Cp levels have been shown in many cardiovascular disorders, including coronary heart disease, myocardial infarction, and arteriosclerosis. The oxidative effects of Cp on serum lipids, in combination with decreased antioxidant protection, can predominate in CAD patients. Ceruloplasmin has diverse functions. It is involved in iron homeostasis and angiogenesis. It is the major source of serum ferroxidase activity and can act as a pro- or antioxidant molecule [37,38,39]. Many previous studies have reported an elevated Cp concentration during HF [40,41]. Some study demonstrated that the Cp can be a significant marker of heart failure in patients with ST segment elevated myocardial infarction [42]. A possible association between ceruloplasmin and progression of HF was study by Cabassi et al. [43].

To the best of our knowledge, only one previous study has evaluated the prognostic value of the simultaneous assessment of Cp and BNP in stable HF patients undergoing elective cardiac evaluation, including coronary angiography. In that study, Hammadah et al. reported that elevated Cp levels increase the risk of 5-year all-cause mortality. Even after adjusting for a large panel of other risk factors and medications, Cp concentration in the third or fourth quartile (> 25.6 mg/dL) remained a significant predictor of increased 5-year mortality. Further analysis, with additional adjustment for heart rate, QRS duration and ICD placement, revealed that a Cp concentration in the upper quartile (> 30.2 mg/dL) remained predictive. Additionally, within each group of defined BNP concentration range, higher Cp levels were associated with poorer outcomes. Similar to our study, the authors shown that the combined use of biomarkers can help identify patients with the highest probability of death [44].

The reasons for the increased Cp concentration in HF are not well understood, but it is possible that the measurement of Cp (in combination with NT-proBNP) can help identify patients with the highest long-term mortality risk.

## 5. Conclusions

The determination of Cp concentration is cost-effective and relatively easy. Data from the present study confirmed the association between Cp concentration and the severity of HF. The combined measurement of Cp and NT-proBNP concentrations has an advantage over measuring NT-proBNP concentration alone in selecting a group of high-risk HF patients in a 1-year follow-up.

### Study Limitations

Our study has several limitations. Firstly, these results may not be applicable to the general population, since the age range of patients in this study was 48–59 years. Secondly, it was a single-center study of stable outpatients considered as potential recipients of the heart. Thirdly, ARNI and SGLT2 were not used, since patient enrollment occurred before 2010. Fourthly, low percentage of patients with implanted ICD.

## Figures and Tables

**Table 1:** Characteristic of the examined group with division according to ceruloplasmin concentration quartiles.

Ceruloplasmin Quartiles (mg/dL)	All Group	I Quartile 8.0–23.6	II Quartile 23.7–28.6	III Quartile 28.7–35.8	IV Quartile 35.9–81.0	
Number	*N* = 741	*N* = 184	*N* = 184	*N* = 187	*N* = 186	
Demographic and clinical parameters						ANOVA
Deaths (*n*)/HT (*n*) All *n* (%)	101/27 128 (17.27)	16/4 20 (10.87)	24/5 29 (15.76)	23/6 29 (15.51)	38/12 50 (26.88)	*p* < 0.001
Female *n* (%)	105 (14.17)	18 (9.78)	25 (13.59)	28 (14.97)	34 (18.28)	NS
Age (years)	54.00 (48.0–59.0)	54.00 (48.00–58.00)	55.00 (49.00–60.00)	54.00 (48.00–59.00)	55.00 (49.00–60.00)	NS
BMI (kg/m^2^)	26.29 (23.50–29.32)	26.49 (24.04–29.06)	26.66 (23.58–29.70)	26.15 (23.36–29.69)	25.96 (22.50–28.89)	NS
Duration of symptoms before inclusion (months)	33.83 (13.07–69.67)	29.82 (13.40–58.47)	33.60 (12.80–69.02)	31.83 (12.90–68.70)	43.77 (14.13–79.93)	NS
Exercise, capacity, echocardiography						
NYHA class III–IV *n* (%)	417 (56.28)	77 (41.85)	99 (53.80)	119 (63.64)	122 (65.59)	*p* < 0.001
VO_2_max (mL/min/kg b.w.)	14.35 (11.70–17.60)	15.30 (12.30–19.50)	14.70 (12.00–17.70)	14.20 (11.40–17.10)	13.40 (10.75–16.55)	*p* < 0.001
LVEF (%)	24.00 (20.00–30.00)	25.00 (20.50–32.50)	24.00 (20.00–30.00)	24.00 22.00–28.00)	22.00 (19.00–28.00)	*p* < 0.01
Laboratory parameters						
NT-proBNP (pg/mL) /100	13.92 (6.44–31.55)	9.30 (5.00–20.09)	14.82 (6.64–34.77)	15.48 (6.55–31.95)	18.42 (8.97–37.96)	*p* < 0.001
Ceruloplasmin (mg/dL)	28.70 (23.70–35.80)	20.75 (18.20–22.40)	26.25 (24.90–27.50)	31.90 (30.00–33.50)	42.35 (38.10–49.30)	*p* < 0.001
Hemoglobin (g/dL)	14.02 (13.05–14.99)	14.02 (13.05–14.83)	14.02 (12.89–14.99)	14.02 (13.22–15.15)	14.18 (13.05–15.15)	NS
Leukocytes (10^9^/L)	6.94 (5.82–8.27)	6.83 (5.53–8.26)	6.77 (5.55–8.27)	7.23 (5.88–8.65)	6.92 (6.07–7.84)	0.060
Blood platelets (10^9^/L)	185.00 (152.00-223.00)	183.00 (148.00-218.50)	185.00 (156.50-220.50)	197.00 (160.00-238.00)	174.00 (150.00-218.00)	*p* < 0.05
Sodium (mmol/L)	136.00 (134.00–139.00)	137.00 (135.00–139.00)	137.00 (134.50–138.00)	135.00 (133.00–138.00)	136.00 (134.00–138.00)	*p* < 0.001
Creatinine clearance (mL/min)	95.11 (69.98–119.44)	101.49 (80.86–125.04)	93.51 (70.86–117.35)	88.85 (70.07–116.43)	93.27 (61.00–117.28)	*p* < 0.01
Uric acid (µmol/L)/10	40.90 (33.00–50.60)	37.85 (33.05–45.00)	41.10 (32.95–50.15)	41.50 (33.10–50.80)	43.25 (32.90–55.60)	*p* < 0.001
Serum protein (g/L)	71.00 (67.00–75.00)	70.00 (66.00–73.50)	70.00 (66.00–74.00)	72.00 (67.00–76.00)	73.00 (69.00–77.00)	*p* < 0.001
Albumin (g/l)	42.00 (39.00–44.00)	42.00 (39.00–44.00)	41.00 (39.00–43.50)	41.00 (38.00–44.00)	43.00 (40.00–45.00)	*p* < 0.05
Fibrinogen (mg/dL)	397.00 (338.00–462.00)	367.00 (320.50–433.50)	395.50 (340.00–454.50)	425.00 (367.00–495.00)	409.50 (343.00–491.00)	*p* < 0.001
C-reactive protein (mg/dL)	2.94 (1.34–6.67)	1.97 (0.91–4.55)	2.65 (1.27–6.04)	4.11 (1.82–7.35)	3.83 (1.86–8.90)	*p* <0.001
Iron concentration (µmol/L)	17.10 (12.00–22.20)	16.91 (13.00–20.25)	17.10 (11.14–21.40)	16.90 (11.80–22.40)	17.60 (12.00–23.92)	NS
Bilirubin (µmol/L)	13.70 (9.70–20.50)	12.00 (8.45–16.15)	13.65 (10.00–18.35)	14.70 (9.30–21.10)	16.55 (11.00–26.60)	*p* < 0.001
Aspartate transaminase (IU/L)	23.0 (19.0–30.9)	23.0 (18.0–29.0)	23 (19.0–28.6)	24 (18.0–310)	24 (20.0–33.0)	NS
Alanine transaminase (IU/L)	24 (17.0–36.0)	23 (17.5–35.5)	24 (17.0–36.0)	24 (17.0–34.0)	25 (18.0–38.0)	NS
γ-glutamyl transpeptidase (IU/L)	49 (27.0–100.0)	39 (24.5–75.5)	45.5 (27.0–79.0)	54 (27.0–112.0)	67.5 (33.0–152.0)	*p* < 0.001
Alkaline phosphatase (IU/L)	68.0 (56.0–90.0)	65.0 (52.0–80.4)	65.0 (54.0–84.0)	72.0 (58.0–94.0)	78.0 (61.0–108.0)	*p* < 0.001
Fasting glucose (mmol/L)	5.50 (5.00–6.20)	5.50 (5.00–6.20)	5.45 (4.85–6.20)	5.60 (5.10–6.70)	5.50 (4.90–6.10)	NS
Total Cholesterol (mmol/L)	4.29 (3.64–5.22)	4.30 (3.60–5.10)	4.25 (3.65–5.19)	4.25 (3.62–5.34)	4.41 (3.67–5.21)	NS
Triglycerides (mmol/L)	1.20 (0.89–1.73)	1.17 (0.83–1.73)	1.22 (0.89–1.93)	1.23 (0.97–1.69)	1.20 (0.85–1.74)	NS
Cholesterol HDL (mmol/L)	1.14 (0.94–1.40)	1.19 (0.98–1.43)	1.14 (0.92–1.39)	1.13 (0.94–1.32)	1.13 (0.88–1.42)	NS
Cholesterol LDL (mmol/L)	2.45 (1.90–3.16)	2.46 (1.89–3.08)	2.39 (1.88–3.20)	2.38 (1.85–3.25)	2.54 (2.00–3.16)	NS
Comorbidities						
Non ischemic DCM; *n* (%)	280 (37.79)	58 (31.52)	77 (41.85)	78 (41.71)	67 (36.02)	NS
Diabetes; *n* (%)	211 (28.48)	43 (23.37)	53 (28.80)	61 (32.62)	54 (29.03)	NS
Arterial hypertension; *n* (%)	408 (55.06)	100 (54.35)	104 (56.52)	91 (48.66)	113 (60.75)	NS
Permanent atrial fibrillation; *n* (%)	176 (23.75)	24 (13.04)	42 (22.83)	48 (25.67)	62 (33.33)	*p* < 0.001
ICD presence; *n* (%)	207 (27.94)	50 (27.17)	63 (34.24)	52 (27.81)	42 (22.58)	NS
Smoker; *n* (%)	257 (34.68)	64 (34.78)	78 (42.39)	72 (38.50)	43 (23.12)	*p* < 0.001
Pharmacotherapy						
Beta-blockers; *n* (%)	726 (97.98)	182 (98.91)	181 (98.37)	180 (96.26)	183 (98.39)	NS
ACE–inhibitors; *n* (%)	641 (86.50)	166 (90.22)	161 (87.50)	159 (85.03)	155 (83.33)	NS
Angiotensin-2 receptor blockers; *n* (%)	76 (10.26)	17 (9.24)	20 (10.87)	24 (12.83)	15 (8.06)	NS
ACE–inhibitor or/and * ARB; *n* (%)	693 (93.52)	178 (96.74)	174 (94,57)	174 (93,05)	167 (89,78)	*p* < 0.05
Loop diuretic; *n* (%)	647 (87.31)	145 (78.80)	168 (91.30)	169 (90.37)	165 (88.71)	*p* < 0.001
Thiazide diuretics; *n* (%)	93 (12.55)	14 (7.61)	19 (10.33)	34 (18.18)	26 (13.98)	*p* < 0.05
Aldosterone receptor antagonist; *n* (%)	683 (92.19)	163 (88.65)	171 (92.93)	177 (94.65)	172 (92.47)	NS
Statins; *n* (%)	487 (65.72)	128 (69.57)	127 (69.02)	124 (66.31)	108 (58.06)	NS
Digitalis; *n* (%)	339 (45.75)	57 (30.98)	82 (44.57)	102 54.55	98 (52.69)	*p* < 0.001

HT: Heart Transplantation; BMI: body mass index; NYHA: New York Heart Association functional class; VO_2_max: maximum oxygen output; LVEF: left ventricle ejection fraction; NT-proBNP: N-terminal pro-B-type natriuretic peptide; HDL: high density lipoproteins; LDL: low density lipoproteins; ICD: Implantable Cardioverter Defibrillator; ACE-inhibitor: angiotensin-converting-enzyme inhibitor; ARB: angiotensin-2 receptor blockers; * (24 patients received ACE-I and ARB simultaneously).

**Table 2:** Characteristic of examined group with division according to ceruloplasmin and N-terminal pro-B-type natriuretic peptide (NT-proBNP) concentration quartiles.

Ceruloplasmin/NT-pro-BNP Quartiles (mg/dL)	I–III Quartile	IV–IV Quartiles	ANOVA
Number	*N* = 683	*N* = 58	
Demographic and clinical parameters			
Deaths (*n*)/HT (*n*) All *n* (%)	79/21 100 (14.64)	23/5 28 (48.28)	*p* < 0.001
Female *n* (%)	96 (14.06)	9 (15.52)	NS
Age (years)	54.00 (49.00–59.00)	56,50 (45,00–61,00)	NS
BMI (kg/m^2^)	26.44 (23.74–29.41)	23.55 (19,86–26.76)	*p* < 0.001
Duration of symptoms before inclusion (months)	33.53 (12.93–68.70)	43.12 (14.3–92.20)	NS
Exercise, capacity, echocardiography			
NYHA class III-IV *n* (%)	369 (54.03)	48 (82.76)	*p* < 0.001
VO_2_max (mL/min/kg b.w.)	14.50 (11.70–18.00)	12.30 (9.20–14.50)	*p* < 0.001
LVEF (%)	24.00 (20.00-30.00)	20.00 (17.00-24.00)	*p* < 0.001
Laboratory parameters			
NT-proBNP (pg/mL) /100	12.78 (5.97–25.70)	52.34 (41.31–78.06)	*p* < 0.001
Ceruloplasmin (mg/dL)	28.00 (23.40–33.70)	46.30 (38.10–54.30)	*p* < 0.001
Hemoglobin (g/dL)	14.02 (13.05–14.99)	13.62 (12.73–15.15)	NS
Leukocytes (10^9^/L)	6.94 (5.79–8.31)	7.01 (6.17–8.19)	NS
Blood platelets (10^9^/L)	185.00 (152.00–224.00)	185.00 (152.00–219.00)	NS
Sodium (mmol/L)	137.00 (134.00–139.00)	134.00 (132.00–137.00)	*p* < 0.001
Creatinine clearance (mL/min)	96.93 (73.45–120.64)	66.41 (50.32–103.34)	*p* < 0.001
Uric acid (µmol/L)/10	40.80 (32.90–49.50)	44.40 (33.70–69.00)	*p* < 0.01
Serum protein (g/L)	71.00 (67.00–75.00)	71.50 (67.00–77.00)	NS
Albumin (g/L)	42.00 (39.00–44.00)	40.00 (38.00–44.00)	*p* < 0.05
Fibrinogen (ug/mL)	396.00 (337.00–458.00)	434.00 (360.00–536.00)	*p* < 0.01
C-reactive protein (mg/dL)	2.80 (1.27–6.12)	7.18 (2.67–14.75)	*p* < 0.001
Iron concentration (µmol/L)	17.20 (12.10–22.20)	16.15 (10.50–21.30)	NS
Bilirubin (µmol/L)	13.40 (9.50–19.30)	22.90 (13.80–32.50)	*p* < 0.001
Aspartate transaminase (IU/L)	23.00 (18.00–30.00)	27.00 (21.00–37.00)	*p* < 0.01
Alanine transaminase (IU/L)	24.00 (17.00–36.00)	25.00 (18.00–41.00)	NS
γ-glutamyl transpeptidase (IU/L)	47.00 (27.00–92.00)	133.50 (49.00–218.00)	*p* < 0.001
Alkaline phosphatase (IU/L)	67.00 (55.00–88.00)	99.50 (73.00–143.00)	*p* < 0.001
Fasting glucose (mmol/L)	5.50 (5.00–6.30)	5.20 (4.70–5.90)	*p* < 0.05
Total Cholesterol (mmol/L)	4.31 (3.66–5.27)	3.97 (3.33–3.86)	NS
Triglycerides (mmol/L)	1.22 (0.89–1.75)	1.07 (0.78–1.36)	*p* < 0.05
Cholesterol HDL (mmol/L)	1.15 (0.95–1.40)	1.05 (0.79–1.29)	*p* < 0.05
Cholesterol LDL (mmol/L)	2.46 (1.91–3.19)	2.29 (1.85–3.00)	NS
Comorbidities			
Non ischemic DCM; *n* (%)	252 (36.90)	28 (48.27)	NS
Diabetes; *n* (%)	190 (27.82)	21 (36.21)	NS
Arterial hypertension; *n* (%)	382 (56.93)	20 (34.48)	*p* < 0.01
Permanent atrial fibrillation; *n* (%)	155 (22.69)	21 (36.21)	*p* < 0.05
ICD presence; *n* (%)	192 (28.11)	15 (25.86)	NS
Smoker; *n* (%)	241 (35.29)	16 27.59)	NS
Pharmacotherapy			
Beta-blockers; *n* (%)	668 (97,80)	58 (100,00)	NS
ACE–inhibitors; *n* (%)	595 (87,12)	46 (79.310	NS
Angiotensin-2 receptor blockers; *n* (%)	71 (10,40)	5 (79,31)	NS
ACE–inhibitors or/and ARB; *n* (%)	643 (94.14)	50 (86,21)	*p* < 0.05
Loop diuretic; *n* (%)	590 (86.38)	57 (98.28)	*p* < 0.05
Thiazide diuretics; *n* (%)	79 (11.57)	14 (24.14)	*p* < 05
Aldosterone receptor antagonist; *n* (%)	628 (91,95)	54 (93,10)	NS
Statins; *n* (%)	457 (66.91)	30 (51.72)	*p* < 0.05
Digitalis; *n* (%)	305 (44.66)	34 (58.62)	NS

HT: Heart Transplantation; BMI: body mass index; NYHA: New York Heart Association functional class; VO_2_max: maximum oxygen output; LVEF: left ventricle ejection fraction; NT-proBNP: N-terminal pro-B-type natriuretic peptide; HDL: high density lipoproteins; LDL: low density lipoproteins; ICD: Implantable Cardioverter Defibrillator; ACE-inhibitors: angiotensin-converting-enzyme inhibitor; ARB: angiotensin -2 receptor blockers.

**Table 3:** Predictors of death or heart transplantation in one-year follow-up. The results of uni- and multivariable Cox regression analysis, model-2.

	Univariable Cox Regression			Multivariable Cox Regression		
	HR	95%CI	*P*	HR	95%CI	*P*
General characteristics						
BMI ↑ (1 kg/m^2^)	0.945	0.908–0.985	*p* < 0.01	0.966	0.912–1.022	NS
Duration of symptoms before inclusion ↑ (1month)	1.004	1.000–1.007	*p* < 0.05	1.000	0.996–1.004	NS
NYHA class ↑ (1 class)	2.936	2.280–3.779	*p* < 0.001	1.099	0.759–1.592	NS
VO_2_max ↓ (1 mL/min/kg b.m.)	1.198	1.142–1.256	*p* < 0.001	1.113	1.048–1.181	*p* < 0.001
LVEF ↓ (1 %p)	1.091	1.059–1.122	*p* < 0.001	1.069	1.032–1.106	*p* < 0.001
Basic biochemistry						
Sodium ↓ (1 mmol/L)	1.111	1.070–1.155	*p* < 0.001	1.039	0.990–1.092	NS
Creatinine clearance ↓ (1 mL/min)	1.014	1.008–1.019	*p* < 0.001	1.001	0.993–1.008	NS
Albumin ↓ (1 g/L)	1.068	1.026–1.114	*p* < 0.01	1.023	0.966–1.083	NS
Cholesterol HDL ↓ (1 mmol/L)	1.805	1.121–2.907	*p* < 0.05	0.954	0.591–1.593	NS
Cp and NT-proBNP ”both in top quartile” (yes/no)	4.253	2.795–6.471	*p* < 0.001	2.120	1.233–3.646	*p* < 0.01
Fibrinogen ↑ (1 mg/dL)	1.003	1.001–1.004	*p* < 0.001	1.001	1.000–1.003	NS
Uric acid ↑ (10 µmol/L)	1.030	1.018–1.041	*p* < 0.001	1.012	0.999–1.026	NS
Bilirubin ↑ (1 µmoL/L)	1.028	1.018–1.039	*p* < 0.001	0.994	0.976–1.012	NS
Alkaline phosphatase ↑ (1 U/L)	1.006	1.004–1.009	*p* < 0.001	1.000	0.995–1.006	NS
γ-Glutamyl trans peptidase ↑ (1 U/L)	1.001	1.000–1.002	*p* < 0.05	1.000	0.998–1.002	NS
Comorbidities						
Diabetes t.2 (yes/no)	1.604	1.123–2.291	*p* < 0.01	1.450	0.949–2.217	NS
ICD absence (yes/no)	9.929	3.922–20.000	*p* < 0.001	7.575	3.278–17.502	*p* < 0.001
Pharmacotherapy						
Lack of ACE - I or/and ARB (yes/no)	3.428	2.126–5.256	*p* < 0.001	2.195	1.234–3.906	*p* < 0.01
Loop diuretics (yes/no)	4.895	1.809–13.248	*p* < 0.01	1.735	0.525–5.730	NS
Thiazide diuretics (yes/no)	2.296	1.518–3.473	*p* < 0.001	1.317	0.781–2.221	NS
Statins (yes/no)	0.699	0.492–0.993	*p* < 0.05	1.294	0.825–2.032	NS
Digitalis (yes/no)	1.439	1.016–2.036	*p* < 0.05	0.833	0.547–1.267	NS

BMI: body mass index; NYHA: New York Heart Association functional class; VO_2_max: maximum oxygen output; LVEF: left ventricle ejection fraction; NT-proBNP: N-terminal pro-B-type natriuretic peptide; Cp: ceruloplasmin, ICD: Implantable Cardioverter Defibrillator; ACE-I: angiotensin-converting-enzyme inhibitor; ARB: Angiotensin-2 receptor blocker.

**Table 4:** Probability of death or heart transplantation occurrence in 1-year follow-up.

	I-III Quartiles of Cp (mg%) (8.0–35.8)	Top Quartile of Cp (mg%) (35.9–81.0)	I-III Quartiles of NT-proBNP (pg/mL) (122.9–3155.0)	Top Quartile of NTpro-BNP (pg/mL) (3156.0–22378.0)	I-III Quartiles of Cp or NT-proBNP	Cp and NT-proBNP Both in Top Quartile
End point (+) (*n*)	78	50	66	62	100	28
End point (−) (*n*)	477	136	490	123	583	30
Probability of end point (%) with confidence intervals	14.054 (11.159–16.941)	26.881 (20.508–33.251)	11.871 (9.182–14.558)	33.513 (26.708–40.312)	14.641 (11.989–17.291)	48.276 (35.416–61.136)
Odds ratio	2.248 95%CI (1.503–3.364) *p* < 0.001		3.742 95%CI (2.511–5.578) *p* < 0.001		5.441 95%CI (3.117–9.498) *p* < 0.001	
Sensitivity (%)	26.88		33.51		48.28	
Specificity (%)	77.81		79.93		95.10	

Cp—ceruloplasmin; NT-proBNP—N-terminal Type B pro peptide.
